# Processing-Route Dependence and Adhesion of Polymer–Adhesive–Steel Interfaces with Recycled PA6/GF30 Composites Modified with Functional Additives: Injection Molding Versus Large-Format Fused Granulate Fabrication

**DOI:** 10.3390/ma19194053

**Published:** 2026-09-22

**Authors:** Robert E. Przekop, Miłosz Frydrych, Natalia Kubiak, Krzysztof Nowak, Bogna Sztorch, Daria Pakuła, Julia Głowacka, Magdalena Kustosz, Tomasz Grabarkiewicz, Jiří Kafka, Peter Scholz

**Affiliations:** 1 Center for Advanced Technologies, Adam Mickiewicz University in Poznań, 61-614 Poznań, Poland; frydrych@amu.edu.pl (M.F.); natalia.kubiak@amu.edu.pl (N.K.); kr.nowak@amu.edu.pl (K.N.); bogna.sztorch@amu.edu.pl (B.S.); daria.pakula@amu.edu.pl (D.P.); julia.glowacka@amu.edu.pl (J.G.); magdalena.kustosz@amu.edu.pl (M.K.); tomasz.grabarkiewicz@amu.edu.pl (T.G.); 2 Fraunhofer Institute for Machine Tools and Forming Technology IWU, Reichenhainer Strasse 88, 09126 Chemnitz, Germany; jiri.kafka@iwu.fraunhofer.de (J.K.); peter.scholz@iwu.fraunhofer.de (P.S.)

**Keywords:** recycled PA6, glass fiber, fused granulate fabrication, large-format additive manufacturing, injection molding, functional additives, pull-off

## Abstract

Large-scale additive manufacturing (LSAM) using fused granulate fabrication (FGF) offers a potential route for the high-value utilization of recycled thermoplastic composites. This study investigated recycled PA6 containing nominally 30 wt.% glass fibers as a candidate feedstock for large-format FGF. An unmodified reference and formulations containing SF315/PTFE, SF315/U15, and MIX-OH were examined. The granulates were characterized using melt mass flow rate measurements, thermogravimetric analysis, and differential scanning calorimetry, while mechanical properties were compared after injection molding and FGF processing. The best FGF performance was obtained for SF315/PTFE (polytetrafluoroethylene) and SF315/U15. Their tensile strengths reached 117.3 and 118.7 MPa, respectively, compared with 56.8 MPa for the FGF-processed reference. Flexural strengths reached 158.8 and 152.4 MPa, respectively, versus 88.0 MPa for the reference. The tensile and flexural improvements produced by MIX-OH after injection molding were not retained after FGF processing, confirming strong processing-route dependence. Preliminary pull-off screening demonstrated that TPU-bonded polymer–steel joints could be produced. Under the investigated conditions, SF315/PTFE and SF315/U15 were identified as promising recycled PA6/GF30 formulations for large-format FGF and further tooling-oriented development.

## 1. Introduction

Fiber-Reinforced Plastic (FRP) composites have become increasingly important engineering materials owing to their unique combination of low density, high mechanical strength, corrosion resistance, and design flexibility [1,2,3]. These advantages have led to their widespread adoption across a broad range of industries, including aerospace [4], automotive [5], marine [6], construction [7], and renewable energy [8]. Consequently, the market for tooling used in FRP part production has grown significantly over the past decades, driven by increasing demand from sectors seeking lightweight and durable alternatives to conventional materials. The continuous growth in the use of high-quality fabricated FRP advanced composite components has led to an intense search for alternative materials and techniques to achieve high-performance, cost-effective tools with high dimensional accuracy and excellent surface quality for moulding composite components. In parallel, advances in composite manufacturing technologies, such as Resin Transfer Molding (RTM) and Vacuum-Assisted Resin Infusion (VARI), have imposed increasingly stringent requirements on mold performance [9,10]. The broad range of FRP applications has created a growing year-by-year demand for both standardized and highly customized tooling solutions [11].

Traditional mold fabrication methods present significant challenges in terms of accommodating design changes reported during product development and production phases. Consequently, the increasing implementation of advanced molding technologies has stimulated innovation in the mold manufacturing sector [12]. Common tooling materials, including metals and composites, provide the dimensional accuracy, thermal stability, and durability required for advanced molding processes and repeated processing cycles without compromising final product quality. However, their manufacturing requires expensive and time-consuming machining and post-processing, leading to high material waste [13,14]. These limitations become particularly significant in prototyping, customized manufacturing, and low-volume production, where tooling expenditure may account for a substantial proportion of the total production cost. The problem becomes even more clear for large and geometrically complex molds, whose fabrication requires considerable quantities of raw material and extensive machining operations.

To address these challenges, Large Scale Additive Manufacturing has attracted considerable attention as an alternative route for mold fabrication. Compared with conventional subtractive fabrication methods, Additive Manufacturing (AM) enables substantial reductions in material waste, production time, and tooling costs [15,16]. In addition, LSAM (Large Scale Additive Manufacturing) simultaneously offers wider design freedom, with fast implementation of design modifications and validation of mold concepts and manufacturing strategies as an extension of traditional additive manufacturing [17,18]. Among the available techniques, direct pellet extrusion, commonly referred to as Fused Granular Fabrication, has attracted considerable interest in tooling applications because it eliminates the need for filament production and enables the processing of highly filled polymer feedstocks [19]. Its high deposition rates and relatively low material costs make fused granular fabrication (FGF) suitable for the efficient fabrication of large-scale polymer structures [20]. The suitability of the technology to the use of recycled polymer feedstocks may further enhance the sustainability of the 3D-printing process by reducing virgin material consumption and lowering the associated environmental burden [21].

Polyamide 6 reinforced with glass fibers (PA6/GF) is extensively employed in the automotive [22], electrical [23], and mechanical engineering [24] sectors due to its favorable mechanical properties, resulting in the generation of considerable quantities of post-industrial waste, including production scrap, rejected components, and processing residues. Post-industrial PA6/GF waste can be mechanically recycled and reused as FGF feedstock, thereby reducing waste, virgin material consumption, and tooling costs [25]. However, mechanical recycling may lead to deterioration of the mechanical and technological properties of PA6/GF due to fiber attrition [26], polymer chain scission [27], and weakening of the fiber–matrix interface during repeated processing cycles [28], necessitating material modification to restore the performance required for tooling application to avoid manufacturing defects in the further processing steps. Low-level organosilicon additives and solid lubricants can modify matrix–fiber interactions, internal friction, and melt behavior [29].

This study aimed to evaluate the suitability of a recycled PA6/GF30 composite for large-scale additive manufacturing using the FGF process through the modification of its properties with organosilicon additives, a coupling agent, and micronized PTFE (polytetrafluoroethylene). The effects of these additives on the mechanical, rheological, and thermal properties of the material were investigated, and the retention of the modification effects after transitioning from injection molding to FGF processing was assessed. In addition, the feasibility of joining polymer and metal components using thermoplastic polyurethane adhesives was evaluated. The ultimate goal was to identify formulations suitable for the production of large-scale, partially recyclable composite tooling structures.

## 2. Materials and Methods

### 2.1. Materials and Formulations

The PA6/GF30 (glass fiber 30% wt.%, Tarnamid T27 GF30 FR VO HF GY 7012 (Grupa Azoty S.A., Tarnów, Poland), constituting waste from public transport seats) feedstock was supplied by Ster Sp. z o.o. (Swadzim, Poland) and is recovered from public-transport seating as recycled material.

BRB SF 315, an amino-functional polydimethylsiloxane fluid (kinematic viscosity: 1500 cSt at 25 °C; nitrogen content: 0.3 wt.%), was supplied by BRB International B.V. (Ittervoort, The Netherlands). DEUREX F 6008 M, a micronized polytetrafluoroethylene (PTFE) powder, was obtained from DEUREX AG (Elsteraue, Germany). The modifier denoted as MIX-OH was prepared by blending BRB OH Fluid 80,000 cSt and BRB OH Fluid 70 cSt, two α,ω-silanol-terminated polydimethylsiloxanes with nominal kinematic viscosities of 80,000 and 70 cSt at 25 °C, respectively, supplied by BRB International B.V. (Ittervoort, The Netherlands), at a mass ratio of 1:1. The organofunctional silane U-15: *N*-(2-aminoethyl)-3-aminopropyltrimethoxysilane was supplied by UNISIL Sp. z o.o. (Tarnów, Poland). All materials were used as received without further purification.

### 2.2. Drying, Compounding, Injection Molding, and FGF

Granulates were dried in a Shini CD-9-CE dryer at 60 °C for 24 h. Components were weighed on a Radwag WLC 30/F1/R balance, premixed for 60 min in a Bitux MS-17198 mixer, and compounded on a GAMART PSHJ-35 twin-screw extruder. The seven-zone PA6 profile was 150/225/230/235/240/240/235 °C, and the screw-speed range was 18–24 rpm. Strands were cooled and pelletized.

Injection-molded type 1A tensile specimens were produced using an Engel E-victory 170/80 injection molding machine (ENGEL, Schwertberg, Austria) with the following zone temperatures: nozzle 215 °C, Zone 1 220 °C, Zone 2 225 °C, Zone 3 215 °C, and feeder 40 °C. The mold temperature was set at 85 °C. Subsequently, type 1B specimens suitable for three-point bending and Charpy impact testing were cut from the type 1A specimens.

3D-printed specimens were fabricated using a two-stage procedure combining Fused Granulate Fabrication (FGF) with abrasive waterjet cutting. FGF printing was carried out using a Stäubli TX200 industrial robotic arm (Stäubli, Pfäffikon, Switzerland) equipped with a CEAD S25 single-screw pellet extruder (CEAD B.V., Delft, The Netherlands, 4 mm nozzle diameter). The feedstock consisted of 30 wt.% short glass fiber-reinforced polyamide 6 (PA6GF30) granules with a nominal particle size of ~3 mm. Prior to processing, the granules were dried in a hot-air dryer at 80 °C for 12 h to achieve a target moisture content below 0.05 wt.%.

Thin-walled blanks with a closed square cross-section were manufactured using a single-perimeter continuous loop deposition path. The extruder barrel zones were heated to set temperatures ranging from 230 °C to 255 °C, with the nozzle set to 260 °C. The extrudate melt temperature measured at the nozzle exit using a calibrated thermal camera was 245 °C. Printing was performed at a linear velocity of 3000 mm/min with a constant mass throughput of 3.0 kg/h. Deposition was conducted onto an unheated aluminum substrate platform kept at ambient room temperature (23 ± 2 °C) under passive air cooling without auxiliary forced airflow.

The printed single-wall blanks had an extruded bead width of 5.0 mm and a nominal layer height of 2.0 mm, achieving uniform layer cooling and structural stability with a reported warpage value of 0.1 mm per blank. After cooling to room temperature, the four corner sections were removed to separate the blank into four flat plates. Tensile and flexural test specimens were subsequently extracted from these plates by abrasive waterjet cutting aligned parallel to the deposition direction (0° orientation relative to extrusion path). With the exception of thickness, which was defined by the wall thickness, the overall specimen dimensions complied with standard test geometries. The gauge area of the extracted mechanical test specimens comprised five deposited layers.

The obtained systems and their compositions are presented in Table 1.

### 2.3. Analytical Methods

Tensile and flexural tests were performed using an Instron 5969 universal testing machine with a maximum load capacity of 50 kN. Flexural testing followed ISO 178 [30] at 2 mm/min to a maximum recorded deflection of 6 mm, and tensile tests followed PN-EN ISO 527 [31]. The crosshead speed was 5 mm/min for tensile testing and 2 mm/min for flexural testing. The Charpy impact tests were performed using an Instron Ceast 9050 instrument according to PN-EN ISO 179-1 [32].

Thermogravimetry (TGA) was performed using a NETZSCH 209 F1 Libra gravimetric analyzer (NETZSCH-Gerätebau GmbH, Selb, Germany). Samples of 5 ± 0.2 mg were placed in Al_2_O_3_ crucibles. Measurements were conducted under nitrogen (flow of 20 mL/min) in the range of 30–1000 °C and 10 °C/min heating rate.

Differential scanning calorimetry (DSC) was performed using a NETZSCH204 F1 Phoenix calorimeter. Samples of 4–7 mg were placed in an aluminum crucible with a punctured lid. Two heating and cooling cycles were performed under nitrogen within a temperature range of 30–250 °C, at heating and cooling rates of 10 °C min^−1^. No cold-crystallization exotherm was observed during either heating scan. Therefore, no correction for cold-crystallization enthalpy was applied. The degree of crystallinity was calculated separately for the first and second heating scans according to Equation (1):

$$X_{c}=\frac{∆H_{m}}{∆H_{m}^{0}\times \omega_{PA6}}\times 100\%$$

where $X_{c}$ denotes the crystallinity degree of the PA6 matrix, $\Delta H_{m}$ is the melting enthalpy obtained from the DSC thermogram, $\Delta H_{m}^{0}$ represents the heat of fusion of fully crystalline PA6 (230 J g^−1^), and $w_{PA6}$ corresponds to the mass fraction of PA6 in the analyzed sample. For neat PA6, $w_{PA6}=1$, whereas for composites and blends the value was corrected according to the actual PA6 content in the formulation.

The melt mass-flow rate (MFR) was measured using an Instron Ceast MF20 plastometer according to ISO 1133 [33]. Measurements were performed at 250 °C/2.16 kg.

In accordance with the respective ISO standards, seven specimens were initially tested for each material formulation and processing condition. Following the standard guidelines, extreme values (outliers) and defective breaks were discarded, resulting in a final sample size of five valid measurements (n = 5) per group. A two-way analysis of variance (ANOVA) was conducted on the mechanical properties. The material formulation and processing route (IM vs. FGF) were considered as fixed factors. Statistical significance was established at α = 0.05.

### 2.4. Pull-Off Adhesion Screening

Two polyurethane hot-melt adhesives were evaluated: DTF TPU Elastic Powder (‘Elastic’) and URET-8020 DTFL—API Polyurethane Powder (‘API’). To conduct the pull-off tests, samples were prepared based on fittings previously produced using 3D printing. Using a CNC cutting machine, disc-shaped samples with a diameter of 20 mm—corresponding to the diameter of the dolly-type components used in the test—were cut from the fittings. The cut discs were bonded to the dolly components using Araldite^®^ two-component epoxy adhesive. After applying the adhesive and joining the two components, the specimens were left for 24 h to ensure complete curing of the adhesive and to achieve a stable bond between the specimen surface and the dolly component.

The prepared samples were tested. The measurement procedure was as follows: the samples for the pull-off test were prepared by preheating the surface of the steel plate to 80 °C. The adhesive was then applied evenly to the surface of the sample. The adhesive-coated sample was then placed on a heated steel plate and pressed against the surface for 5–10 s to ensure proper contact and distribution of the adhesive layer. After assembly, the bonded specimens were left under ambient conditions for 30 min to allow complete curing of the adhesive. Pull-off strength was measured with a DeFelsko PosiTest AT-A electronic hydraulic adhesion tester (DeFelsko Corporation, Ogdensburg, NY, USA), with a pull rate of 0.7 MPa/s. The adhesive layer thickness was 0.85 ± 0.04 mm. The sample diameter was 20 mm corresponding to a nominal bonded area of 314 mm^2^. The entire specimen surface was in contact with the steel plate during bonding; therefore, the nominal circular area was considered the effective bonded area for calculation of the pull-off strength.

All adhesive joints were prepared using the same heat-activated bonding procedure described above. The tests were performed on the interfaces between PA6-based material specimens and a steel plate. For each material tested, 7 measurements were taken. The results were used to determine the average and standard deviation. All measurements were performed at room temperature.

## 3. Results and Discussion

### 3.1. Melt Flow Rate (MFR)

The MFR value of the unmodified PA6/GF30 composite was 37.1 g/10 min. The incorporation of modifying additives resulted in a reduction in this parameter, with the largest decrease observed for the SF315/PTFE system, for which the MFR was 23.3 g/10 min, corresponding to a 37.2% reduction relative to the reference material. For the SF315/U15 and MIX-OH composites, MFR values of 26.1 and 30.7 g/10 min were obtained, representing decreases of 29.6% and 17.3%, respectively (Table 2).

The reduction in MFR indicates an increase in the flow resistance of the molten composites. This effect may be attributed to changes in the interactions between the PA6 matrix, glass fibers, and modifying additives, as well as to variations in the dispersion state of the individual constituents within the material. In the case of the SF315/U15 composite, interactions between amino and alkoxysilane groups and the amide groups of PA6, as well as hydroxyl groups present on the glass fiber surface, may play a significant role. Such interactions may lead to restricted macromolecular mobility and improved interfacial adhesion.

It is noteworthy that the lowest MFR values were obtained for the SF315/PTFE and SF315/U15 composites, which also exhibited the best mechanical performance after FGF processing. In contrast, the MIX-OH composite, which showed the smallest reduction in MFR, did not demonstrate a comparable improvement in properties following the transition from injection molding to FGF processing. These findings suggest that there is no simple relationship between melt flowability and the quality of additively manufactured components. Although increased flow resistance may contribute to improved stability of the deposited bead, the final mechanical performance of printed parts is also governed by factors such as interlayer bonding, fiber orientation, and the material’s thermal history.

### 3.2. Thermal Properties

Thermogravimetric analysis (TGA) and differential scanning calorimetry (DSC) were performed on the obtained granulates. Based on the TG and DTG curves, the temperature corresponding to a 5% mass loss (T_5%_), the temperature of the maximum mass-loss rate (T_max_), and the residue at 1000 °C after analysis under a nitrogen atmosphere were determined (Figure 1).

The TG and DTG curves of all the investigated materials show one main mass-loss stage associated with the degradation of the PA6 matrix [34]. For some samples, weak secondary effects were also recorded above 500 °C. The T_5%_ value increased from 353.5 °C for the reference PA6/GF30 to 388.0–390.7 °C for the modified materials. However, this increase was not accompanied by a comparable shift in T_max_. For MIX-OH and SF315/PTFE, the T_max_ values were 457.0 and 456.8 °C, respectively, and were close to the value obtained for the reference material (457.6 °C). This means that the modified materials reached 5% mass loss at a higher temperature, while the position of the maximum of the main PA6 degradation stage remained practically unchanged. Thus, the increase in T_5%_ indicates a delay in the initial mass loss but cannot be interpreted as an improvement in the thermal stability of the composites. A different degradation behaviour was recorded for the SF315/U15 system. Despite the highest T_5%_ value of 390.7 °C, T_max_ decreased to 449.7 °C, i.e., by 7.9 °C relative to the reference PA6/GF30. The SF315/U15 system delayed the attainment of 5% mass loss, whereas the dominant matrix degradation stage occurred at a lower temperature and was characterized by a higher maximum mass-loss rate. The opposite directions of the changes in T_5%_ and T_max_ indicate a modification of the degradation behaviour rather than a uniform increase in the thermal stability of the material.

The pyrolytic residue was 26.50% for the reference PA6/GF30 and 28.82–30.41% for the modified materials. It consisted mainly of glass fibers and small amounts of carbonaceous residue and inorganic products formed from the organosilicon additives [35].

The applied modifying systems primarily affected the initial degradation behaviour of PA6/GF30. MIX-OH and SF315/PTFE increased T_5%_ without causing a significant change in T_max_, whereas SF315/U15 combined an increase in T_5%_ with a decrease in T_max_. Therefore, the increase in T_5%_ should be interpreted as a delay in reaching 5% mass loss rather than as an unequivocal increase in the thermal stability of the material over the entire temperature range.

The melting (T_m_) and crystallization (T_c_) temperatures were determined based on the positions of the maxima of the corresponding thermal effects, whereas the melting (ΔH_m_) and crystallization (ΔH_c_) enthalpies were determined by integrating the peak areas (Figure 2, Table 3). The thermograms of all the investigated materials showed a single dominant melting effect characteristic of the crystalline phase of PA6 [36]. No distinct cold-crystallization effect was recorded during heating. In the first heating cycle, the T_m_ values ranged from 223.9 to 225.4 °C, whereas in the second cycle they ranged from 221.0 to 221.7 °C.

The lower T_m_ values recorded in the second cycle are consistent with the elimination of the previous thermal history. The small differences in melting temperature between the individual systems indicate that the applied additives did not substantially affect the position of the main PA6 melting peak. The melting enthalpy determined during the first heating cycle was clearly dependent on the material composition and ranged from 41.75 to 45.68 J g^−1^. After the elimination of thermal history, the range of ΔH_m_ values shifted to 35.46–39.27 J g^−1^. This result suggests that the differences between the two heating cycles were partly related to the morphology of the crystalline phase formed during compounding and previous processing.

The most pronounced change in the crystallization behaviour was observed for the SF315/U15 system. The crystallization peak temperature of this material was approximately 4.1 °C lower than that of the unmodified PA6/GF30 during the first cooling cycle.

The decrease in T_c_ means that the crystallization peak of the modified SF315/U15 system occurred at a greater degree of supercooling than in the reference material. This result is consistent with a delay in the crystallization process, potentially associated with restricted mobility of the PA6 segments [37]. This change may be associated with interactions of the amino and alkoxysilane groups with the matrix components and the glass-fiber surface. For the SF315/PTFE and MIX-OH systems, the T_c_ values remained close to those obtained for the reference material. For SF315/PTFE, weak endothermic effects were also recorded at approximately 56.1 and 106.4 °C during the first heating cycle. The first may be associated with enthalpy relaxation in the glass-transition region of PA6, whereas the second may be related to the release of moisture or other volatile components. The absence of analogous effects during the subsequent heating cycle is consistent with their association with the initial thermal history or volatile content of the material.

The applied additives did not cause significant changes in the melting temperature of PA6. Their influence was primarily evident in the melting enthalpy and crystallization behaviour. For the SF315/U15 system, a decrease in crystallization temperature was observed during both cooling cycles, indicating a change in the kinetics of this process. The degree of crystallinity calculated from the first heating cycle was 25.93% for PA6/GF30, 27.55% for SF315/PTFE, 28.96% for SF315/U15, and 26.60% for MIX-OH. In the second heating cycle, the corresponding values were 24.39%, 22.75%, 22.47%, and 23.90%, respectively. The narrower range of crystallinity values obtained during the second cycle indicates that the differences observed during the first heating cycle were partly associated with the previous thermal history of the granulates. The results also show that crystallinity alone cannot explain the differences in mechanical performance between the formulations.

### 3.3. Mechanical Response After Injection Molding and Large-Format FGF

#### 3.3.1. Tensile Properties

The tensile properties differed considerably between the injection-molded and FGF-printed materials (Figure 3A–C). For the unmodified reference PA6/GF30, the tensile strength decreased from 77.5 ± 8.2 MPa after injection molding to 56.8 ± 5.5 MPa after FGF processing. The corresponding tensile modulus decreased from 4461.3 ± 347.5 to 2209.6 ± 240.5 MPa.

After injection molding, the incorporation of all the investigated modifiers increased both tensile strength and tensile modulus. Relative to the injection-molded reference, tensile strength increased by 50.5% for SF315/PTFE, 13.2% for SF315/U15, and 39.7% for MIX-OH. The tensile modulus increased to 5425.4 ± 380.1 MPa for SF315/PTFE, 4974.7 ± 175.1 MPa for SF315/U15, and 5403.1 ± 138.3 MPa for MIX-OH, corresponding to increases of 21.6%, 11.5%, and 21.1%, respectively.

Following large-format FGF processing, the tensile response was strongly dependent on the formulation. Relative to the printed reference, the tensile strength and tensile modulus of SF315/PTFE increased by 106.5% and 120.4%, respectively. Similar improvements were observed for SF315/U15, for which tensile strength increased by 109.0% and tensile modulus by 91.2%. In contrast, MIX-OH did not improve tensile strength after FGF processing, while its tensile modulus was 21.8% lower than that of the printed reference.

A direct comparison of the corresponding formulations processed using the two technologies demonstrates the different stability of the modification effects. For SF315/PTFE, FGF processing changed tensile strength by only +0.6% relative to the injection-molded formulation, although the tensile modulus decreased by 10.2%. For SF315/U15, FGF processing increased tensile strength by 35.3%, despite a 15.1% reduction in tensile modulus. In contrast, the MIX-OH formulation exhibited reductions of 47.6% in tensile strength and 68.0% in tensile modulus after FGF processing relative to the corresponding injection-molded material.

The effect of the processing route was also evident in elongation at break. For the injection-molded materials, elongation at break decreased from 7.0% for the reference PA6/GF30 to 5.5% for SF315/PTFE and 6.0% for both SF315/U15 and MIX-OH. These changes correspond to decreases of 21.4%, 14.3%, and 14.3%, respectively. Thus, the improvement in tensile strength and stiffness after injection molding was accompanied by lower deformability.

For the FGF-printed materials, elongation at break remained within the relatively narrow range of 6.0–7.0%. It increased from 6.0% for the printed reference to 7.0% for SF315/PTFE, remained at 6.0% for SF315/U15, and reached 6.3% for MIX-OH. Accordingly, the large increases in tensile strength and stiffness of the SF315-containing printed materials were not accompanied by a loss of deformability.

These formulation-dependent responses can be related to the combined effects of the additives on the existing PA6–glass-fiber interphase, melt flow, and the microstructure generated during secondary processing. The amino-functional SF315 may interact with the carbonyl groups of PA6 and with polar sites available at the glass-fiber surface, thereby modifying fiber wetting and local stress transfer during recompounding and subsequent specimen formation.

In SF315/U15, the amino groups of U15 may additionally interact with the amide groups of PA6, while its alkoxysilane groups may exhibit affinity toward accessible hydroxyl-containing sites on the glass surface [38]. Because U15 was introduced directly into recycled PA6/GF30 during melt recompounding, this effect should be interpreted as an in situ modification of an existing interphase rather than as conventional silane pretreatment of the glass fibers [39].

#### 3.3.2. Charpy Impact Strength

Charpy impact strength depended strongly on both formulation and processing route (Figure 3D). For the injection-molded reference PA6/GF30, the impact strength was 49.5 ± 4.6 kJ m^−2^. Following modification, this value was 42.0 ± 2.0 kJ m^−2^ for SF315/PTFE, 48.2 ± 4.2 kJ m^−2^ for SF315/U15, and 43.4 ± 3.1 kJ m^−2^ for MIX-OH. The above data show decreases of 15.2%, 2.6%, and 12.3%, respectively, compared to the reference value for injection-molded samples. Thus, under injection-molding conditions, the modifiers reduced the capacity of the materials to absorb energy under impact loading, with the smallest decrease observed for SF315/U15.

An opposite trend was observed for the FGF-printed materials. The impact strength of the printed reference PA6/GF30 was 23.9 ± 4.2 kJ m^−2^. Following modification, values of approximately 37.5 ± 4.1 kJ m^−2^, 36.9 ± 4.9 kJ m^−2^, and 30.1 ± 3.9 kJ m^−2^ were obtained for SF315/PTFE, SF315/U15, and MIX-OH, respectively. These values correspond to increases of 57.1%, 54.6%, and 26.0% relative to the printed reference. The improvement in impact strength was therefore most pronounced for the formulations containing SF315.

When it comes to direct comparison of the two processing routes, the results demonstrate that the impact performance of the samples was dependent on the processing method and type of additive. For all investigated formulations, the impact strength of the FGF-printed specimens was lower than that of the corresponding injection-molded specimens. This difference may be attributed to the distinct mesostructure produced by additive manufacturing, including imperfect interlayer bonding and process-induced voids. However, the effect of the additives varied markedly between the two processing routes. In the injection-molded specimens, the incorporation of the additives resulted in a reduction in impact strength relative to the reference material, indicating a formulation-dependent change in the fracture response. In contrast, the same modifying systems substantially improved the impact resistance of the FGF-printed specimens. This opposite response highlights the strong dependence of the modification effect on the processing route. This finding suggests that they may enhance interlayer adhesion or possibly cause better layer-to-layer remelting or otherwise modify the fracture behaviour of the printed structures, but it needs to be further investigated for a better understanding of the phenomenon behind this.

In SF315/PTFE, PTFE most likely acted primarily as a solid lubricant affecting internal friction, melt flow, and processing-induced fiber arrangement rather than as a reinforcing phase. Previous studies of PTFE-containing PA6/GF composites reported a limited direct contribution of PTFE to tensile strength but a pronounced effect on tribological behavior and, depending on concentration, impact resistance [40].

#### 3.3.3. Flexural Properties

The flexural properties also showed pronounced differences between the two processing routes (Figure 4A,B). For the injection-molded reference PA6/GF30, the flexural modulus and flexural strength were 4065.3 ± 252.1 MPa and 87.2 ± 4.5 MPa, respectively. For the FGF-printed reference, the corresponding values were 2952.3 ± 319.1 MPa and 88.0 ± 8.5 MPa. Thus, FGF processing reduced the flexural modulus of the unmodified material, whereas its flexural strength remained similar to that of the injection-molded reference.

For the injection-molded SF315/PTFE formulation, the flexural modulus and flexural strength increased to approximately 7053.5 and 151.7 MPa, respectively. These values correspond to increases of 73.5% and 74.0% relative to the injection-molded reference. After FGF processing, the flexural modulus and flexural strength of SF315/PTFE increased by 102.3% and 80.5%, respectively, relative to the printed reference. A direct comparison with the corresponding injection-molded formulation shows that FGF processing reduced its flexural modulus by 15.3%, while its flexural strength was retained and increased slightly by approximately 4.7%.

For the injection-molded SF315/U15 formulation, the flexural modulus and flexural strength increased by 19.5% and 16.3%, respectively, relative to the injection-molded reference. After FGF processing, the corresponding increases relative to the printed reference were substantially greater and amounted to 97.7% and 73.2%. Compared with the injection-molded SF315/U15 formulation, FGF processing increased the flexural modulus by 20.2% and the flexural strength by 50.3%.

MIX-OH produced a markedly different response. After injection molding, this formulation exhibited increases of 68.3% in flexural modulus and 64.7% in flexural strength relative to the injection-molded reference. No corresponding improvement was observed after FGF processing: the flexural modulus and flexural strength were 2.7% and 1.1% lower, respectively, than those of the printed reference. Relative to the corresponding injection-molded MIX-OH formulation, FGF processing reduced the flexural modulus by 58.0% and the flexural strength by 39.4%.

The considerably greater increases in the flexural properties of the injection-molded SF315/PTFE and MIX-OH materials than in their tensile moduli indicate that the response of these formulations depended on the loading mode. Processing-induced fiber arrangement, surface-layer morphology, and local stress transfer within the PA6–glass-fiber interphase may have contributed to this behavior [41].

The loss of the MIX-OH-related improvement after FGF processing indicates that its favorable effect on flexural properties was strongly dependent on the processing route. In contrast, the FGF-printed SF315/PTFE and SF315/U15 formulations exhibited higher tensile and flexural moduli and strengths than the printed reference. The observed contrast shows that the mechanical response to FGF processing was strongly formulation dependent. For improved visualization of the trends and relative differences in mechanical performance, the percentage changes in individual mechanical parameters after FGF processing relative to the corresponding injection-molded formulation are summarized in a heat map (Figure 5).

The mechanical property responses were statistically evaluated to determine the significance of variations introduced by the additive modifications and the manufacturing process. A two-way ANOVA performed on the central property dataset confirmed that the interaction between the processing route (IM vs. FGF) and the formulation is highly statistically significant (*p* < 0.001) for tensile strength, tensile modulus, and impact strength. Both main factors (processing method and additive type) also exhibited significant statistical impacts (*p* < 0.001). The strong interaction effect directly validates that the modifying mechanisms are fundamentally processing-route dependent—a formulation that enhances a specific property under injection molding does not necessarily yield an equivalent improvement during FGF (and vice versa). While changes in elongation at break showed less statistical prominence due to relatively high inherent variances (overlapping error bars), the overarching trends in strength and stiffness robustly substantiate the process-specific behavior of the modified systems.

### 3.4. Room-Temperature TPU Pull-Off Screen

To evaluate the adhesive properties of the PA6-based material-adhesive–steel interface systems, pull-off tests were conducted (Table 4, Figure 6). For the DTF TPU Elastic Powder adhesive, the adhesion strength values obtained for PA6-based material–adhesive–steel interfaces ranged from 0.29 to 0.54 MPa. For PA6/GF30, a value of 0.54 ± 0.07 MPa was obtained, which was the highest value obtained for this adhesive type. For modified PA6/GF30 material–adhesive–steel interfaces, the values were 0.29 ± 0.03 MPa for PA6/GF30 + SF315/U15, 0.39 ± 0.007 MPa for PA6/GF30 + SF315/PTFE, and 0.40 ± 0. 0.05 MPa for PA6/GF30 + MIX-OH.

For the URET-8020 DTFL—API Polyurethane Powder adhesive, higher pull-off strength values were obtained than for the Elastic adhesive for all modified PA6 material–adhesive–steel interfaces. The values ranged from 0.48 to 0.63 MPa. For PA6/GF30, the value was 0.52 ± 0.06 MPa. The highest value was obtained for PA6/GF30 + MIX-OH, for which the average adhesion strength was 0.63 ± 0.03 MPa. For the other modified PA6/GF30 material–adhesive–steel interfaces, the values obtained were 0.48 ± 0.03 MPa for PA6/GF30 + SF315/U15 and 0.50 ± 0.02 MPa for PA6/GF30 + SF315/PTFE.

A comparison of the adhesive systems used shows that URET-8020 DTFL—API Polyurethane Powder provided higher adhesive strength in the pull-off test for all analyzed modified PA6-based material interfaces. The greatest difference was observed for the PA6/GF30 + MIX-OH–adhesive–steel interface for which the use of the API adhesive increased the average pull-off stress from 0.40 to 0.63 MPa.

In all investigated cases, adhesive failure was observed during the pull-off tests. After separation, the adhesive layer remained on the surface of the PA6-based specimen, while the steel plate was largely free of adhesive. This indicates that the failure occurred predominantly at the adhesive–steel interface, rather than within the adhesive layer or at the adhesive–polymer interface.

## 4. Conclusions

The conducted research demonstrated that recycled PA6/GF30 can be transformed into a fully functional engineering material suitable for large-scale additive manufacturing. Appropriate modification of the composition made it possible to mitigate the loss of properties associated with the change in processing technology and, in the case of selected formulations, to achieve mechanical properties significantly superior to those of the unmodified reference material. The most promising formulations were SF315/PTFE and SF315/U15. After FGF processing, their tensile strength was more than twice that of the unmodified PA6/GF30 produced by the FGF method, while flexural strength increased by 80.5% and 73.2%, respectively. For both formulations, an increase in impact strength of more than 50% was also recorded compared with the printed reference material. These results indicate that a small amount of suitably selected additives can substantially improve the mechanical performance of recycled PA6/GF30 after FGF processing.

The obtained results demonstrate the potential for high-value utilization of post-industrial PA6/GF30 composites instead of downcycling them into applications with lower performance requirements. The use of the developed formulations in the production of large-scale tooling could reduce the consumption of virgin polymers, extend the service life of both the PA6 matrix and the embedded glass fibers, and decrease the material intensity associated with mold manufacturing.

Preliminary investigations of polymer adhesive–steel joints further indicate the potential application of the developed materials in the construction of hybrid molds. However, full validation of their application potential requires verification of material properties in independent printing campaigns, evaluation of mold durability under service conditions, and assessment of property retention after successive cycles of grinding and reprocessing.

## Figures and Tables

**Figure 1:**
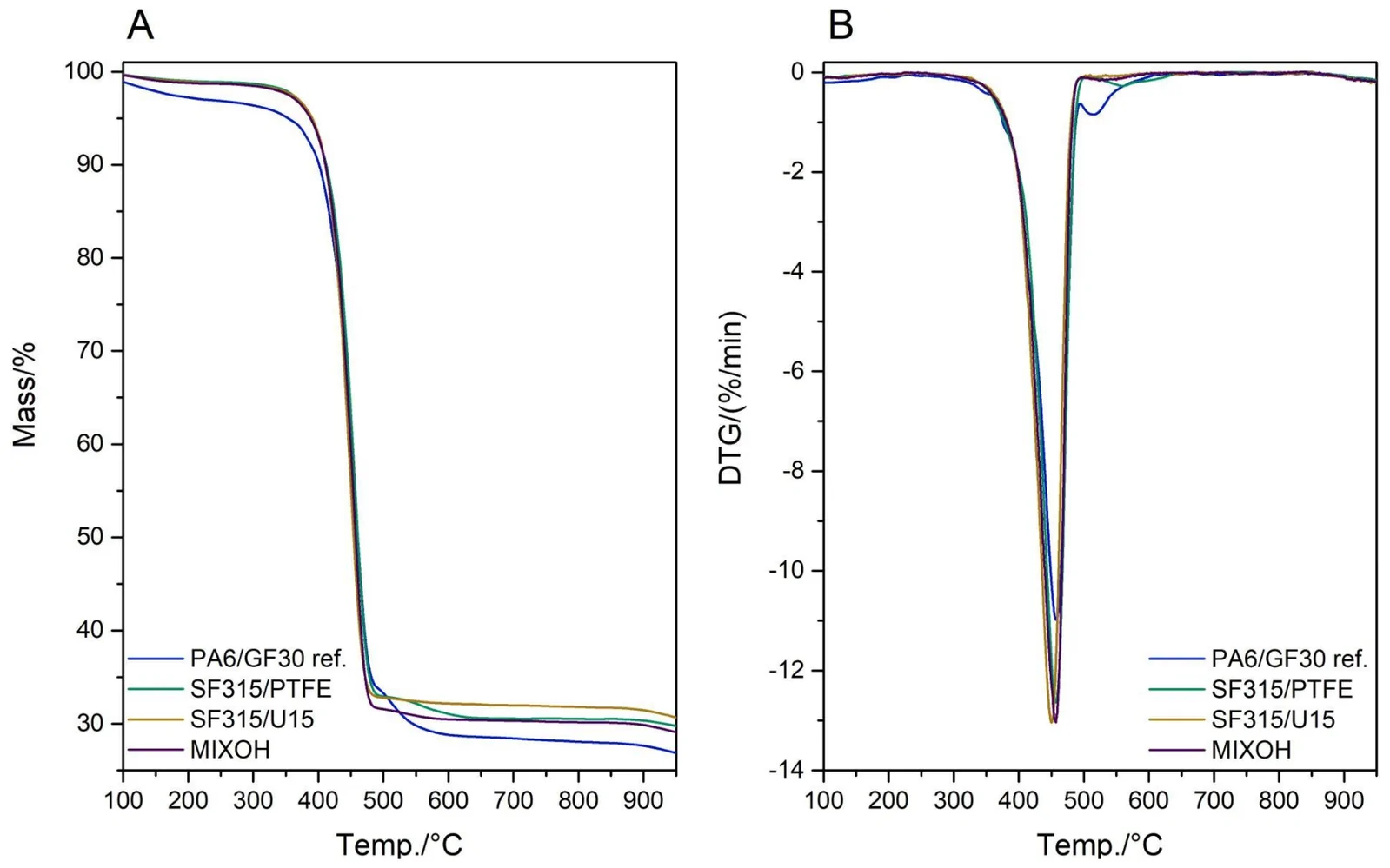
TGA (**A**) and DTG (**B**) curves of the reference PA6/GF30 material and the SF315/PTFE-, SF315/U15-, and MIX-OH-modified composites, recorded under a nitrogen atmosphere at a heating rate of 10 °C min^−1^.

**Figure 2:**
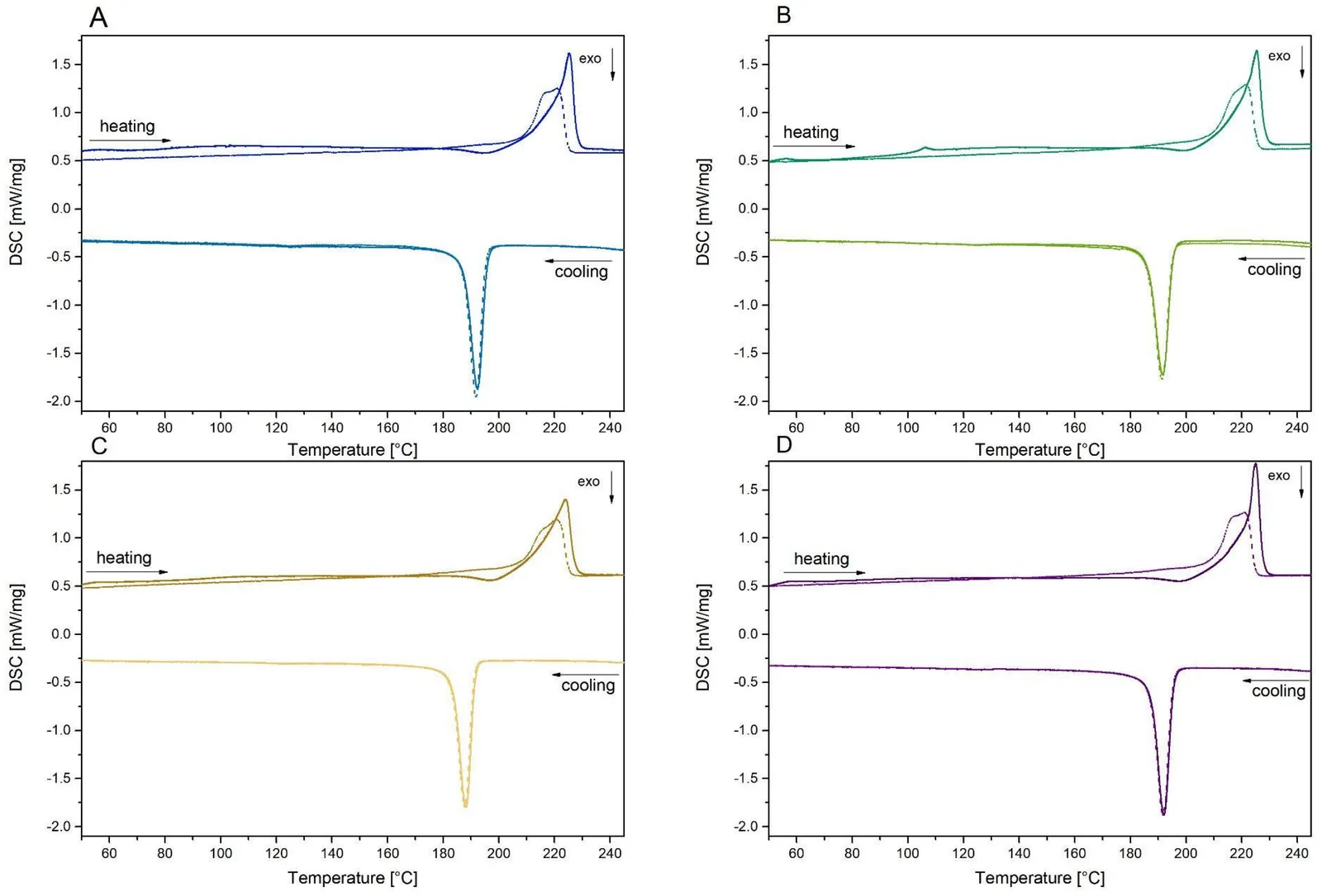
DSC curves recorded during the first and second heating and cooling cycles for: (**A**) PA6/GF30, (**B**) PA6/GF30/SF315/PTFE, (**C**) PA6/GF30/SF315/U15, and (**D**) PA6/GF30/MIX-OH. Solid lines correspond to the first cycle, and dashed lines to the second cycle. The direction of the exothermic effect is indicated in the plots.

**Figure 3:**
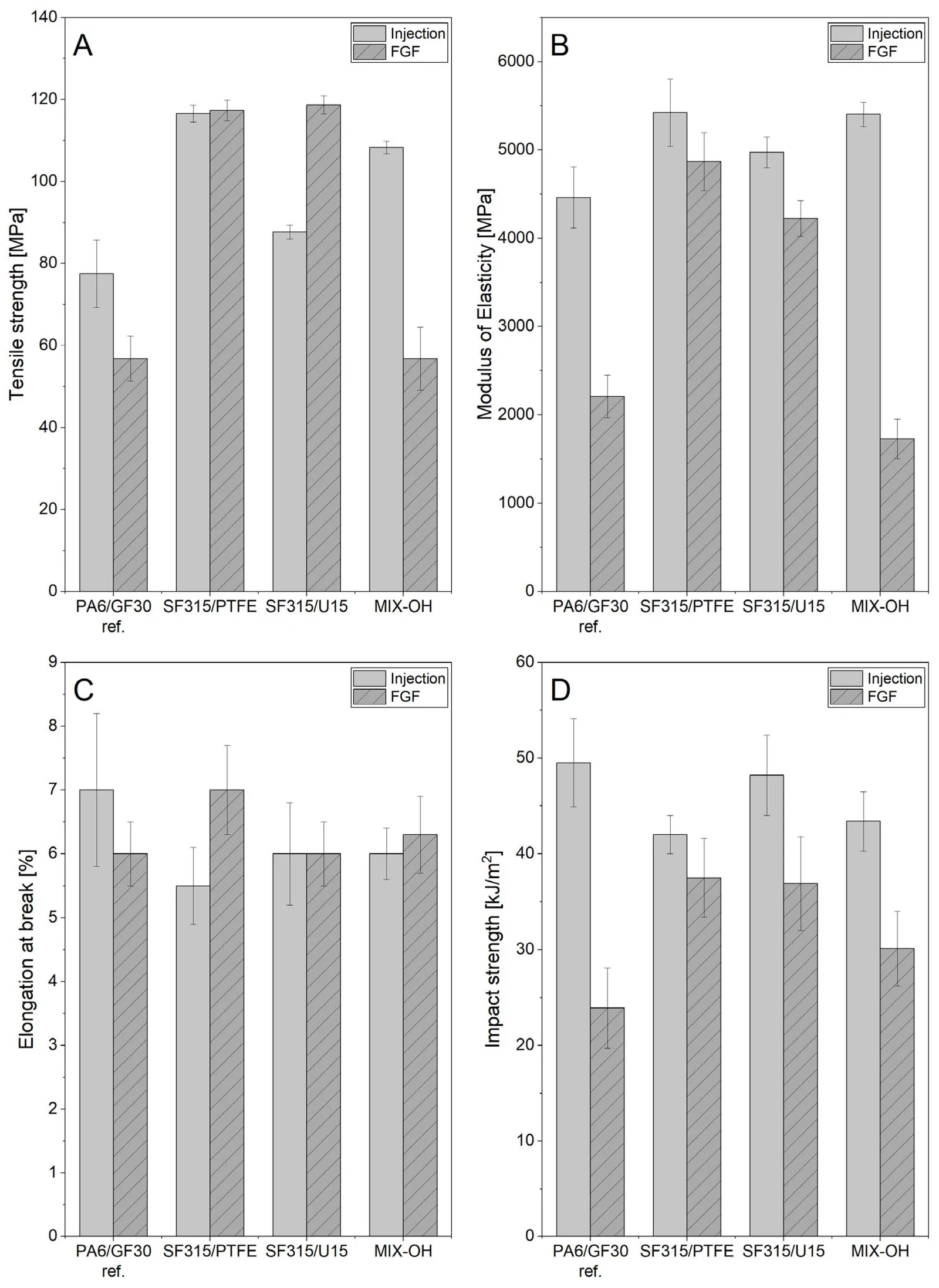
Mechanical properties of the reference PA6/GF30 and modified PA6/GF30 systems (SF315/PTFE, SF315/U15, and MIX-OH) processed by injection molding and fused granulate fabrication (FGF): (**A**) tensile strength, (**B**) tensile modulus, (**C**) elongation at break, and (**D**) impact strength.

**Figure 4:**
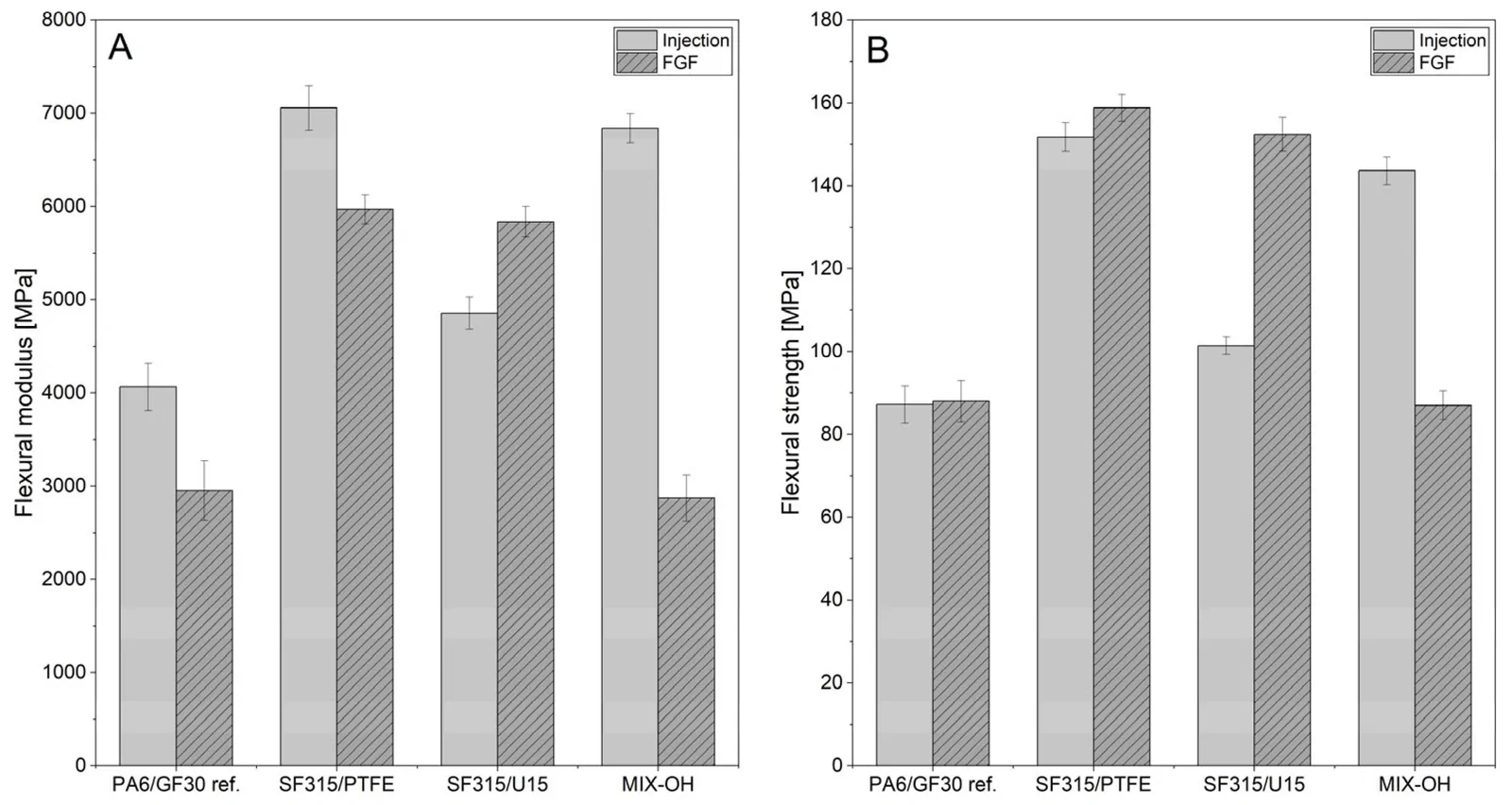
Flexural properties of the reference PA6/GF30 and modified PA6/GF30 systems (SF315/PTFE, SF315/U15, and MIX-OH) processed by injection molding and fused granulate fabrication (FGF): (**A**) flexural modulus and (**B**) flexural strength.

**Figure 5:**
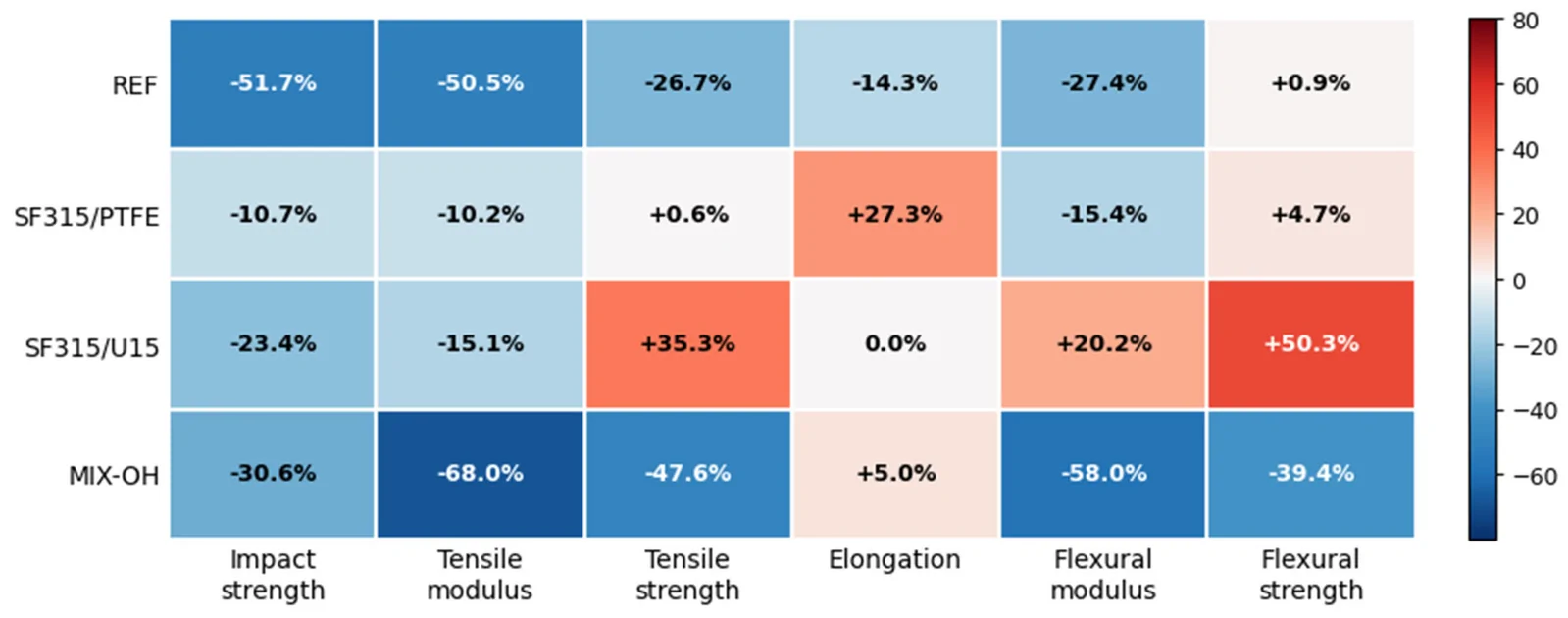
Heat map of FGF change relative to the matching injection-molded formulation.

**Figure 6:**
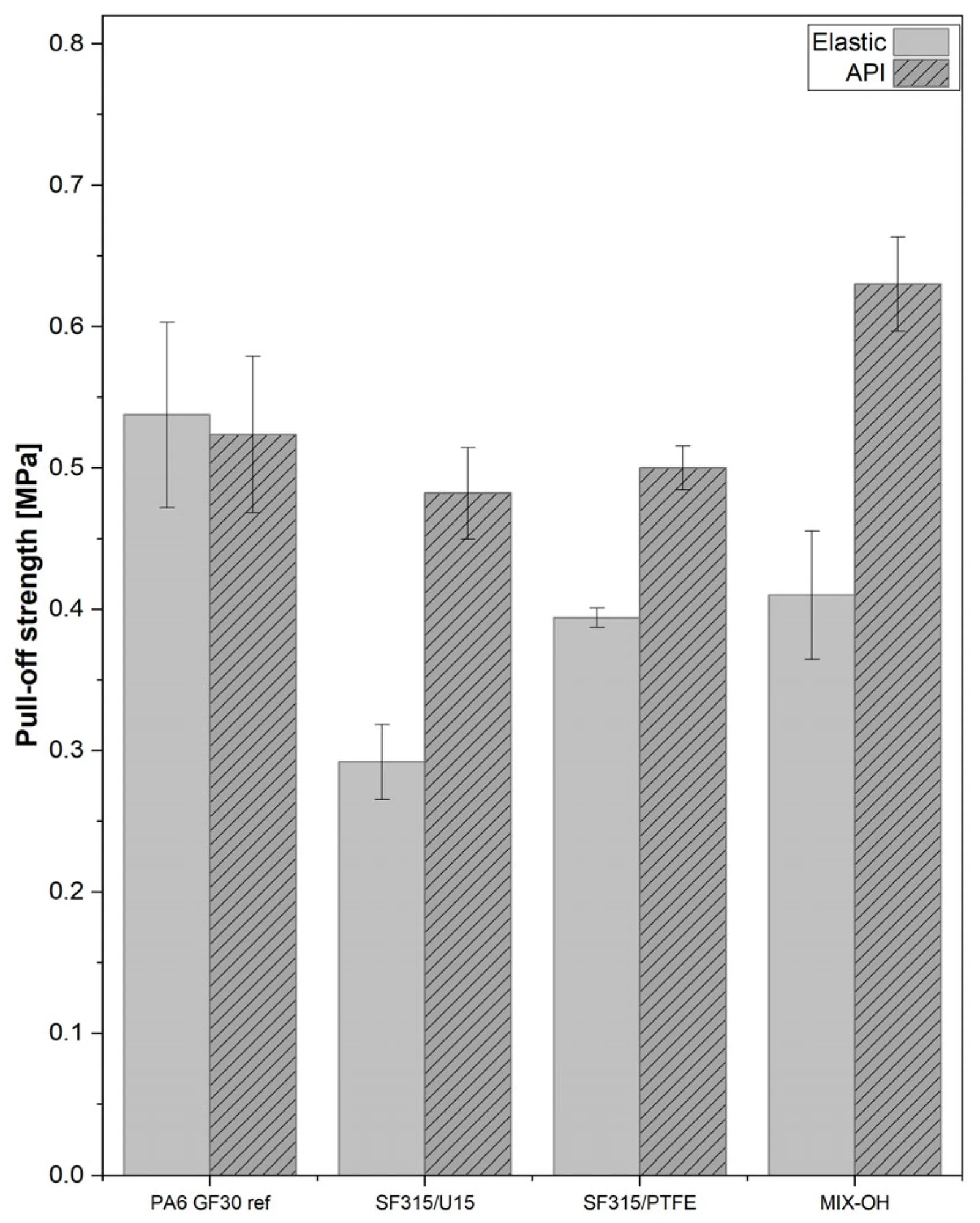
Room-temperature pull-off strength on steel.

**Table 1:** PA6/GF30 formulations used in the injection molding–FGF comparison.

Short Code	Base Feedstock	Additives
REF	Recycled PA6, nominal 30 wt.% GF	None
SF315/PTFE	Recycled PA6, nominal 30 wt.% GF	1 wt.% SF315 + 1 wt.% PTFE
SF315/U15	Recycled PA6, nominal 30 wt.% GF	1 wt.% SF315 + 1 wt.% U15
MIX-OH	Recycled PA6, nominal 30 wt.% GF	2 wt.% MIX-OH Fluid 80,000/70

**Table 2:** Melt-flow analysis.

Formulation	MFR (g/10 min)
REF	37.1
SF315/PTFE	23.3
SF315/U15	26.1
MIX-OH	30.7

**Table 3:** TGA and DSC analysis.

	TGA Analysis			DSC Analysis									
Sample	T_5%_ [°C]	T_max_ [°C]	Residue [%]	T_m1_ * [°C]	ΔH_m1_ * [J/g]	T_m2_ ** [°C]	ΔH_m2_ ** [J/g]	T_c1_ * [°C]	ΔH_c1_ * [J/g]	T_c2_ ** [°C]	ΔH_c2_ ** [J/g]	*X*_c1_ *[%]	*X*_c2_ ** [%]
REF	353.5	457.6	26.50	225.2	41.75	221.0	39.27	192.3	−47.63	191.8	−47.87	25.93	24.39
SF315/PTFE	388.2	456.8	29.48	225.4	43.46	221.7	35.90	191.7	−47.40	191.4	−46.55	27.55	22.75
SF315/U15	390.7	449.7	30.41	223.9	45.68	221.0	35.46	188.2	−46.37	187.7	−45.62	28.96	22.47
MIX-OH	388.0	457.0	28.82	225.0	41.98	221.2	37.72	192.1	−47.04	191.8	−46.37	26.60	23.90

* first cycle, ** second cycle; negative ΔH_c_ values indicate exothermic crystallization.

**Table 4:** Room-temperature pull-off strength.

Sample	Elastic [MPa]	API [MPa]
PA6/GF30	0.54 ± 0.07	0.52 ± 0.06
PA6/GF30 + SF315/U15	0.29 ± 0.03	0.48 ± 0.03
PA6/GF30 + SF315/PTFE	0.39 ± 0.007	0.50 ± 0.02
PA6/GF30 + MIX-OH	0.40 ± 0.05	0.63 ± 0.03

## Data Availability

The original contributions presented in this study are included in the manuscript. Further inquiries can be directed to the corresponding author.
